# From monoclonals to bispecific T cell engagers: the evolving antibody-based therapy landscape in acute myeloid leukemia

**DOI:** 10.3389/fimmu.2026.1803185

**Published:** 2026-06-01

**Authors:** Milad Rasouli, Marie Dalem, Marc H.G.P. Raaijmakers, Peter D. Katsikis, Stefan J. Erkeland

**Affiliations:** 1 Department of Immunology, Erasmus University Medical Center, Rotterdam, Netherlands; 2 Department of Hematology, Erasmus MC Cancer Institute, Rotterdam, Netherlands

**Keywords:** acute myeloid leukemia, antibody, bispecific T cell engager, immunotherapy, leukemic stem cell, relapsed/refractory acute myeloid leukemia

## Abstract

Acute myeloid leukemia (AML) is an aggressive hematological malignancy with a poor prognosis despite advances in treatment strategies. Standard treatment regimens induce remissions in many patients, but relapse occurs in approximately half, highlighting the urgent need for novel therapeutic strategies. Antibody-based therapies have significantly improved the treatment of acute lymphoblastic leukemia (ALL), but progress in AML has been slower, with only gemtuzumab ozogamicin (GO) receiving FDA approval to date. This is largely due to the absence of AML-specific surface antigens and the challenge of distinguishing malignant blasts from normal hematopoietic cells, which raises concerns about on-target/off-leukemia toxicity. Nonetheless, a wide range of antibody constructs, including unconjugated monoclonal antibodies, antibody-drug conjugates, and bispecific T cell engagers (BTCEs), are now under investigation in both preclinical studies and clinical trials, predominantly in the relapsed/refractory (R/R) setting. Encouraging results from early-phase studies suggest that antibody-based approaches could complement or even partially replace traditional cytotoxic regimens in selected patient groups. In this review, we summarize the spectrum of antigenic targets explored for AML immunotherapy, critically assess clinical outcomes achieved so far, and discuss current efforts to improve efficacy, durability, and safety. We also highlight emerging strategies aimed at overcoming antigen heterogeneity and resistance, thereby advancing antibody-based therapies toward broader clinical application in AML.

## 1 Introduction

Acute myeloid leukemia (AML) is a biologically heterogeneous and aggressive hematological malignancy characterized by abnormal clonal expansion of malignant myeloid blasts, which accumulate in the bone marrow (BM) (1, 2). For five decades, the standard treatment for medically fit AML patients, has been intensive induction chemotherapy, most commonly the so-called ‘7 + 3’ regimen followed by consolidation therapy, which may consist of an allogeneic hematopoietic stem cell transplantation (allo-HSCT) (3). This standard approach is often associated with significant toxicity and, despite its intensity, frequently fails to eradicate leukemia-initiating cells (LICs), which can persist even after complete remission and ultimately cause relapse (4). Accordingly, considerable interest in de-intensifying conventional AML therapy has driven the development of targeted approaches, many of which have received regulatory approval since 2017 (5).

Antibody-based therapeutics enable selective targeting of surface antigens and induce cytotoxicity through pro-apoptotic mechanisms or by activating innate immune responses (6). Following its approval in 1997 and significant clinical success, Rituximab, an anti-CD20 monoclonal antibody (mAb) used to treat B-cell lymphomas, paved the way for the development of various other antibody modalities, including bispecific antibodies (BsAbs) and antibody-drug conjugates (ADCs), for the treatment of hematological malignancies (7–9).

This review aims to provide a comprehensive overview of antibody-based therapeutic strategies for AML, with a particular emphasis on bispecific T cell engagers (BTCEs) in preclinical development or under early clinical trial evaluation. We highlight the recent scientific progress and remaining challenges in advancing antibody-based approaches into effective AML treatments.

## 2 Bispecific T cell engagers

Progress in antibody engineering has resulted in the development of BTCEs that possess varied structures and pharmacological properties. Each BTCE consists of two segments, each capable of binding to a distinct antigen (10). BTCEs are broadly divided into two categories based on their structure: immunoglobulin G (IgG)-like BTCEs, and non-IgG-like BTCEs (10, 11). IgG-like BTCEs, due to their Fc fragment and larger size, generally have an extended half-life in the bloodstream and are more resistant to proteolysis. In contrast, non-IgG-like BTCEs, with their smaller size, lack Fc-mediated recycling through the neonatal Fc receptor (FcRn) and therefore have a shorter circulation half-life. Furthermore, their compact structure makes them less immunogenic and enables improved tissue distribution and penetration. This offers advantages for targeting solid tumors or other challenging tissues such as the BM. Larger antibody-based reagents may face limitations in these settings due to restricted diffusion across dense extracellular matrices, impaired penetration through vascular or stromal barriers, and reduced access to leukemic or tumor cells residing within protective microenvironments (12–15).

**Figure 1:**
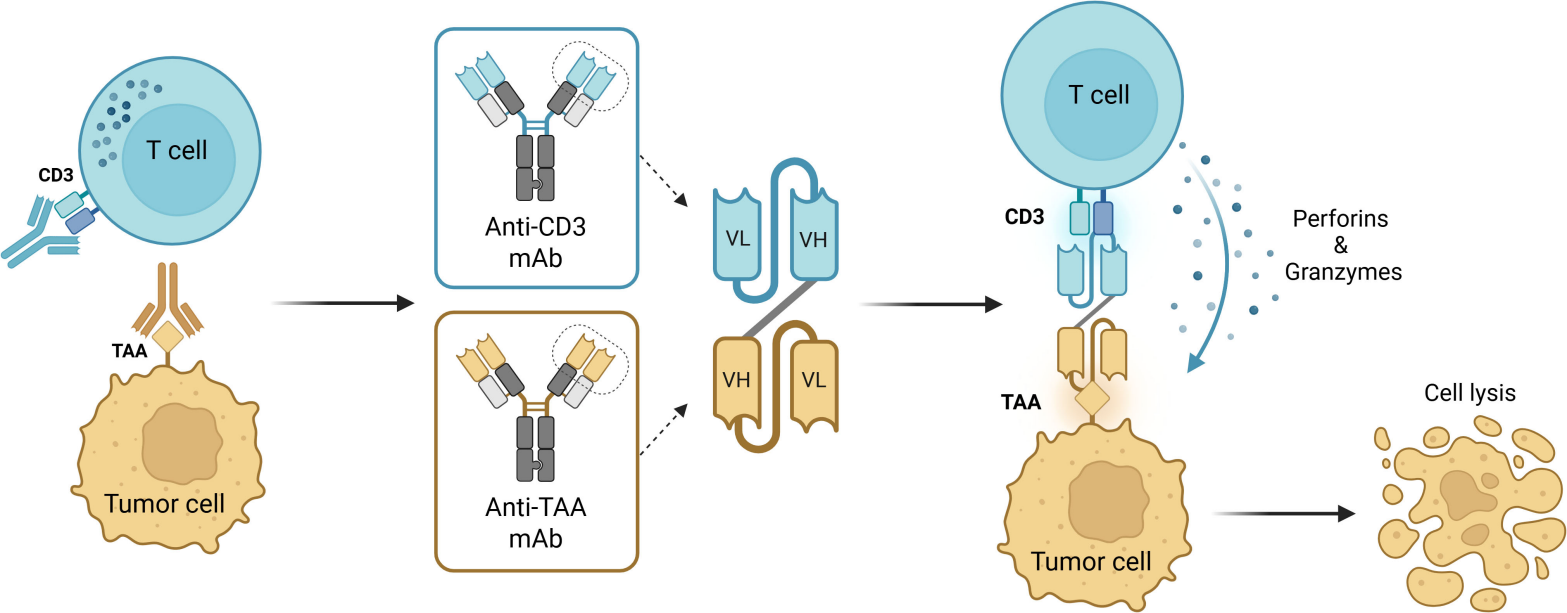
A schematic illustration depicting the structure and mechanism of action of a canonical bispecific T cell engager (BTCE). CD3, cluster of differentiation 3; TAA, tumor-associated antigen; mAb, monoclonal antibody; VH, variable region heavy chain; VL, variable region light chain Created with BioRender.com.

In some settings, where prolonged activity is desired, the short half-life of BTCEs may be a disadvantage. For example, in the case of leukemia or solid tumors where cytotoxicity may be required over days or weeks, a short half-life may impede clinical benefit. Several strategies can be employed to extend the circulation half-life of non-IgG-like BTCEs to maintain sufficient *in vivo* exposure, sustain target engagement, and achieve durable antitumor activity without requiring continuous infusion. These include modifications such as: 1) PEGylation, which involves attaching polyethylene glycol chains to enhance stability, decrease immunogenicity and reduce renal clearance, 2) multimerization through peptide linkers to increase molecular size, and 3) fusion to albumin or albumin-binding domains, which leverages the natural long half-life of albumin for improved pharmacokinetics (16–18). Dual binding of BTCE to leukemic cells and T cells triggers T cell activation independently of T cell receptor (TCR) specificity, ultimately resulting in the destruction of target cells by cytotoxic CD8+ T cells (**Figure 1**). While different BTCEs are being developed and designed to target specific AML-associated antigens, a common feature across nearly all BTCEs is their ability to bind to the CD3 co-receptor signaling complex on T cells. Below, we will discuss the AML-targeting BTCEs that were recently tested in clinical trials and those that are in preclinical development and promising for future clinical testing.

## 3 Chimeric antigen receptor-T cells

Chimeric antigen receptor (CAR)-T cell therapies have revolutionized the treatment of relapsed/refractory (R/R) B-cell malignancies and multiple myeloma by achieving high response rates and durable remissions through targeted redirection of engineered autologous T cells (19, 20). However, despite intensive research, no CAR-T cell product has yet been approved for AML. A major challenge in AML is the is the scarcity of truly leukemia-specific surface antigens.

Most candidate targets, such as CD33, CD123, and CLL-1, are also expressed on normal hematopoietic stem and progenitor cells (HSPCs), raising the risk of severe on-target/off-tumor toxicity, including prolonged or permanent myeloablation (21, 22). CAR-T efficacy in AML is further limited by the highly immunosuppressive tumor microenvironment (TME), which promotes T cell exhaustion and impairs anti-leukemic activity (23, 24). This review does not cover CAR-T cells; for a detailed and comprehensive discussion, readers are encouraged to refer to reviews in this field (25, 26).

Compared to antibody-based strategies (including monoclonal antibodies, antibody-drug conjugates, and bispecific T cell engagers), CAR-T cell treatments offer the advantage of *in vivo* expansion and persistence, enabling long-term immune surveillance and durable remissions (27, 28). However, they require complex *ex vivo* manufacturing processes and are associated with significant toxicities, including cytokine release syndrome (CRS) and immune effector cell-associated neurotoxicity (29, 30), and are logistically challenging to implement. In contrast, antibody-based therapies provide off-the-shelf availability, standardized dosing, and generally more manageable safety profiles (31, 32). While their efficacy may be limited by short serum half-life, reliance on host immune effector cells, and potential development of resistance mechanisms (33, 34), recent advances in half-life extension and multispecific formats are helping to address these limitations.

By offering greater practicality and a potentially wider therapeutic window in AML, antibody-based approaches, particularly BTCEs, have emerged as a central focus in current AML immunotherapy development.

## 4 Target antigens for antibody-based therapies in AML

**Figure 2:**
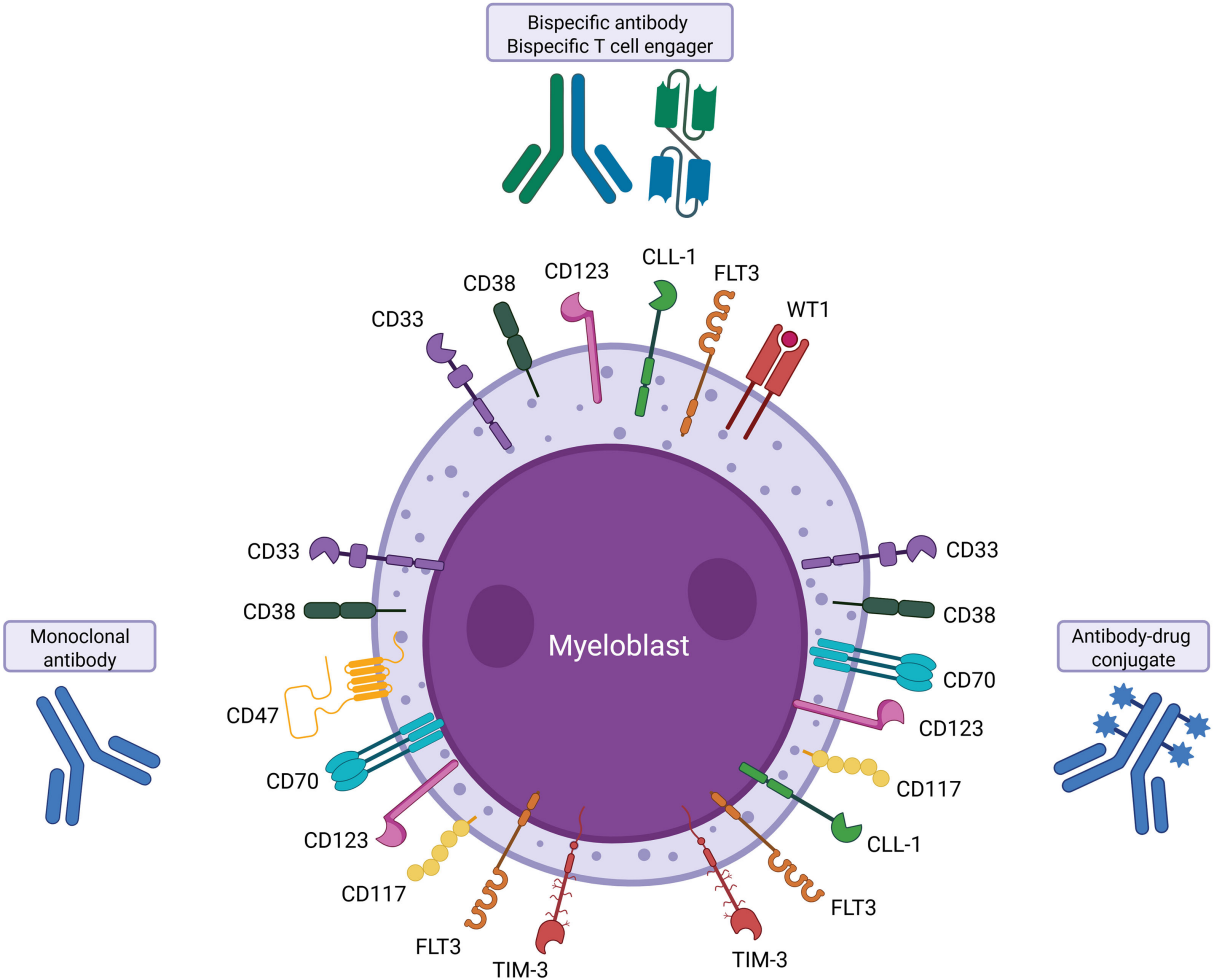
Schematic representation of surface antigens expressed on AML cells that have been clinically investigated for antibody-based therapies, including monoclonal antibodies, bispecific antibodies/T cell engagers, and antibody-drug conjugates. Targets include CD33, CD38, CD47, CD70, CD123, CD117, CLL-1, FLT3, TIM-3, and WT1 Created with BioRender.com.

The ideal antigenic target for antibody-based therapy in AML is one that is AML-specific, meaning it is highly and uniformly expressed on leukemic cells, with minimal or no expression on healthy cells and tissues. Moreover, this target should be highly expressed on LICs, be critical for leukemia cell survival to prevent escape from targeting, and exhibit high immunogenicity. Despite extensive genomic and molecular profiling of AML, no universal antigen target suitable for antibody-based therapies across all AML subtypes has been identified, largely due to the biological heterogeneity of the disease (35). Selected antigen targets in AML can generally be classified as 1) lineage-restricted antigens (LRAs), which are cell-surface antigens confined to the myeloid lineage (e.g., CD33, CD123); 2) leukemia-associated antigens (LAAs), which are overexpressed on AML cells (e.g., FLT3, WT1), and 3) leukemia-specific antigens (LSAs) (e.g., mutated NPM1, mutated FLT3), which are neoantigens formed by leukemia-specific mutations in protein-coding genes (36–38). For this review, we focused on the most extensively studied target antigens for antibody-based targeting of AML (**Figure 2**).

### 4.1 CD33 – lineage-restricted antigen

The transmembrane glycoprotein CD33, also known as sialic acid-binding immunoglobulin-like lectin 3 (SIGLEC-3), is a myeloid differentiation marker found on normal myeloid precursor cells, maturing granulocytes and monocytes. Importantly, CD33 is not expressed outside the hematopoietic system or on multipotent hematopoietic stem cells (HSCs) (39). CD33 is detected on the surface of blasts in approximately 85%–90% of AML patients, though expression levels may vary considerably (40, 41). Because CD33 is often detected on AML blasts and LICs at higher levels than on normal HSCs, it has been widely pursued as an immunotherapy target (42). However, clinical results with CD33-directed agents have been mixed, highlighting both the opportunities and the biological challenges of this approach.

Gemtuzumab ozogamicin (GO) is an ADC consisting of a humanized anti-CD33 mAb covalently linked to the cytotoxic agent N-acetyl-γ-calicheamicin. The antibody selectively binds to CD33 on the cell surface. Upon binding and inducing internalization of CD33, calicheamicin is released intracellularly, where it induces DNA double-strand breaks, ultimately triggering cell death (43). Initially, GO received accelerated approval in 2000 for treatment of AML (44). However, it was subsequently withdrawn from the market in 2010 due to limited clinical benefit and increased early mortality (45). In 2017, GO was reapproved and reintroduced on the market after new data demonstrated that lower, fractionated dosing improved patient outcomes while reducing toxicity (46, 47). In Europe, GO has been available since 2018 and is indicated for the treatment of newly diagnosed CD33-positive (CD33+) AML in patients aged 15 years and older, in combination with daunorubicin and cytarabine (48). As the most studied target antigen for antibody-based therapies in AML and following the success of GO, several agents targeting CD33 have been developed and evaluated in clinical trials, including up to phase-III, but none have achieved regulatory approval (49–56) (**Table 1**).

**Table 1:** Overview of AML-directed antibody-based therapies (mAb and ADC) in clinical trials for AML.

Target	Drug name (format)	Trial phase (NCT)	Indication (trial status)	Treatment	Key findings/conclusion	References
CD33	AVE9633 (ADC)	Phase I (NCT00543972)	R/R CD33+ AML (Terminated)	AVE9633	Terminated due to absence of evidence of clinical activity up to toxic doses	(49)
CD33	BI 836858 (mAb)	Phase I (NCT01690624)	R/R AML & AML in CR with high risk to relapse (Completed)	BI 836858	BI 836858 is safe but has limited efficacy as monotherapy	(50, 51)
		Phase I (NCT03207191)	AML relapse after allo-HSCT (Completed)	BI 836858 + F16IL2	BI 836858 plus F16-IL2 is tolerable with modest anti-leukemic activity	
		Phase I/II (NCT02632721)	AML (Completed)	BI 836858 + decitabine	BI 836858 plus decitabine is tolerable and demonstrates potential anti-leukemic activity in elderly or R/R AML	
CD33	BL-M11D1 (ADC)	Phase I (NCT05924750)	R/R AML (Recruiting)	BL-M11D1	–	–
		Phase I (NCT06714591)	R/R AML (Recruiting)	BL-M11D1	–	
CD33	IMGN779 (ADC)	Phase I (NCT02674763)	R/R CD33+ AML (Completed)	IMGN779	IMGN779 shows some clinical activity in R/R CD33+ AML but overall efficacy is limited	(52)
CD33	Lintuzumab (SGN-33; HuM195) (mAb)	Phase I (NCT00283114)	AML & MDS (Completed)	Lintuzumab	Lintuzumab is well-tolerated and shows some clinical activity, but does not provide a clear survival benefit	(53, 54)
		Phase II (NCT00006084)	AML patients that did not respond to standard treatment in clinical trial PDL 195-301 (Unknown)	Lintuzumab	–	
		Phase II (NCT00528333)	Untreated AML in patients ≥ 60 years (Completed)	Cytarabine + lintuzumab	Lintuzumab combined with low-dose cytarabine does not improve overall response or survival compared to cytarabine alone	
		Phase III (NCT00006045)	R/R AML (Unknown)	Standardized chemotherapy (+lintuzumab)	–	
CD33	Vadastuximab talirine (SGN-CD33A) (ADC)	Phase I (NCT01902329)	CD33+ AML (Completed)	Vadastuximab talirine (+HMA)	Vadastuximab talirine demonstrates activity and a tolerable safety profile as a single agent in patients with AML	(55, 56)
		Phase I (NCT02326584)	Newly diagnosed AML (Completed)	Vadastuximab talirine (+other standard treatments)	Vadastuximab talirine combined with standard chemotherapy is well-tolerated and produces high rates of deep remission	
		Phase I/II (NCT02614560)	R/R AML (Terminated)	Vadastuximab talirine in sequence with allo-HSCT	–	
		Phase III (NCT02785900)	Older patients with newly diagnosed AML (Terminated)	Vadastuximab talirine + azacitidine/decitabine	Terminated due to safety; specifically a higher rate of deaths, including fatal infections, in the SGN33A arm versus the control arm	
CD123	BYON4413 (ADC)	Phase I (NCT06359002)	R/R AML or MDS (Recruiting)	BYON4413	–	–
CD123	CSL360 (mAb)	Phase I (NCT00401739)	R/R/HR-AML (Completed)	CSL360	No results posted	–
CD123	Pivekimab sunirine (IMGN632) (ADC)	Phase I (NCT04086264)	CD123+ AML (Active)	IMGN632 (+venetoclax and/or azacitidine)	Under investigation	–
		Phase I/II (NCT06034470)	Newly diagnosed adverse-risk AML (Recruiting)	Pivekimab sunirine + FLAG-Ida	–	
CD123	SGN-CD123A (ADC)	Phase I (NCT02848248)	R/R AML (Terminated)	SGN-CD123A	–	–
CD123	Talacotuzumab (JNJ-56022473; CSL362) (mAb)	Phase I (NCT01632852)	CD123+ AML in CR or CRi at high risk of early relapse (Completed)	CSL362	Talacotuzumab is well-tolerated in HR-AML patients in remission and effectively depletes CD123+ cells	(57, 58)
		Phase II (NCT02992860)	AML & MDS failing HMA-based therapy (Terminated)	JNJ-56022473	Enrollement was stopped due to a recommendation by the IDMC/FDA for a parallel clinical trial with TALA, where potentially no efficacy could be determined	
		Phase II/III (NCT02472145)	AML in patients unfit for intensive chemotherapy (Completed)	Decitabine (+ talacotuzumab)	Talacotuzumab plus decitabine did not improve outcomes compared to decitabine alone and was associated with increased toxicity	
CD47	CC-90002 (mAb)	Phase I (NCT02641002)	AML and HR-MDS (Terminated)	CC-90002	Preliminary monotherapy data in R/R AML and HR-MDS did not offer a sufficiently encouraging profile for further dose escalation/expansion	(59)
CD47	Gentulizumab (GenSci059) (mAb)	Phase I (NCT05263271)	R/R AML or MDS (Unknown)	Gentulizumab	–	–
CD47	Magrolimab (Hu5F9-G4) (mAb)	Phase I (NCT02678338)	R/R AML and HR-MDS (Completed)	Hu5F9-G4	Magrolimab is generally well-tolerated and shows promising activity, but requires careful monitoring of hemoglobin and blood compatibility	(60–63)
		Phase I (NCT03248479)	(R/R) AML/(HR-)MDS (Terminated)	Magrolimab (+azacitidine)	Discontinued due to futility based on the results of a planned analysis of the ENHANCE trial	
		Phase I (NCT03922477)	R/R AML (Terminated)	Hu5F9-G4 + atezolizumab	The sponsor decided to discontinue the development of atezolizumab in combination with magrolimab in the AML indication	
		Phase I (NCT05823480)	Patients who underwent allo-HSCT for HR-AML/MDS (Withdrawn)	Magrolimab + azacitidine	–	
		Phase I/II (NCT04435691)	AML (Terminated)	Magrolimab + azacitidine + venetoclax	Terminated due to 75% > participants	
		Phase II (NCT05829434)	Newly diagnosed AML or HR-MDS (Withdrawn)	Magrolimab + intensive chemotherapy	Trial was stopped prematurely due to safety reasons	
		Phase III (NCT05079230)	Newly diagnosed AML in patients previously untreated and unfit for intensive chemotherapy (Terminated)	Magrolimab + azacitidine + venetoclax	Terminated due to futility	
		Phase III (NCT04778397)	AML or MDS (Terminated)	Magrolimab + azacitidine	Discountinued due to futility	
CD47	Lemzoparlimab (TJ011133) (mAb)	Phase I (NCT04912063)	AML or MDS (Terminated)	Lemzoparlimab + venetoclax and/or azacitidine	Terminated following strategic considerations	(64)
		Phase I/II (NCT04202003)	AML or MDS (Completed)	Lemzoparlimab (+azacitidine)	Lemzoparlimab is generally well-tolerated and shows preliminary anti-leukemic activity	
CD47	Letaplimab (IBI188) (mAb)	Phase I/II (NCT04485052)	AML (Suspended)	Letaplimab + demethylating agents	Paused for changes in development strategies	–
CD70	Cusatuzumab (ARGX-110) (mAb)	Phase I (NCT04241549)	Newly diagnosed AML or HR-MDS in patients unfit for intensive treatment (Completed)	Cusatuzumab + azacitidine	Cusatuzumab plus azacitidine is well-tolerated and shows preliminary anti-leukemic activity	(65, 66)
		Phase I (NCT04023526)	Newly diagnosed AML in patients unfit for intensive chemotherapy (Active)	Cusatuzumab + azacitidine	Under investigation	
		Phase II (NCT04150887)	AML (Active)	Cusatuzumab + various therapies	Under investigation	
		Phase II (NCT06384261)	Newly diagnosed AML in patients unfit for intensive therapy (Recruiting)	Venetoclax + azacitidine (+cusatuzumab)	–	
		Phase I/II (NCT03030612)	Newly diagnosed AML or HR-MDS (Completed)	Cusatuzumab + azacitidine	Cusatuzumab monotherapy and in combination with azacitidine is highly active. Cusatuzumab eliminates CD70-expressing LSCs potentially leading to deep and durable remissions.	
CD70	SEA-CD70 (ADC)	Phase I (NCT04227847)	AML & MDS (Recruiting)	SEA-CD70 (+azacitidine)	–	–
CLL-1	DCLL9718S (ADC)	Phase I (NCT03298516)	R/R AML or patients previously untreated AML and unfit for intensive induction chemotherapy (Completed)	DCLL9718S (+azacitidine)	DCLL9718S has limited tolerability and anti‐tumor activity	(67)
TIM-3	KK2845 (ADC)	Phase I (NCT06812104)	R/R AML (Recruiting)	KK2845	–	–
TIM-3	Sabatolimab (MBG453) (mAb)	Phase I (NCT03066648)	AML or HR-MDS (Completed)	MBG453 (+PDR001) or PDR001 and/or MBG453 + decitabine/azacitidine	The combination of sabatolimab and decitabine was safe and well tolerated, and exhibited evidence of anti-leukemic activity	(68)
		Phase I (NCT03940352)	AML or HR-MDS (Terminated)	HDM201 + MBG453/venetoclax	Terminated following a business decision	
		Phase I/II (NCT04623216)	MRD+ AML post-allo-HSCT (Terminated)	Sabatolimab (+azacitidine)	Terminated following a strategic decision from the sponsor. It was not based on any safety findings or safety concerns with sabatolimab.	
		Phase I/II (NCT05367401)	AML or MDS (Withdrawn)	Sabatolimab + magrolimab (+azacitidine)	Waiting for update from study lead	
		Phase I/II (NCT06664879)	AML (Recruiting)	MBG-453 + azacitidine	–	
		Phase II (NCT04150029)	AML in patients unfit for chemotherapy (Terminated)	MBG-453 + azacitidine + venetoclax	Terminated because results from 2 trials in the program were negative (including the main trial) and Novartis decided to terminate the program all together	
TIM-3	TQB2618 (mAb)	Phase I (NCT05426798)	R/R AML & MDS (Unknown)	TQB2618 + azacitidine + decitabine	–	–
CD38	Daratumumab (Darzalex^®^; HuMax-CD38; JNJ-54767414) (mAb)	Phase I (NCT05749276)	Adverse risk AML in patients ≥ 60 years (Not yet recruiting)	Daratumumab + chemotherapy (idarubicin & cytarabine)	–	(69)
		Phase I/II (NCT03537599)	Relapsed AML post-allo-HSCT (Terminated)	Daratumumab + DLI	Terminated due to poor accrual	
		Phase II (NCT03067571)	R/R AML or HR-MDS (Terminated)	Daratumumab	Terminated early due to lack of efficacy	
CD38	STI-6129 (ADC)	Phase I (NCT05519527)	R/R T-ALL or AML (Withdrawn)	STI-6129	0 patient accrual	–
FLT3	ASP1235 (AGS62P1) (ADC)	Phase I (NCT02864290)	AML (Terminated)	ASP1235	Study was terminated due to lack of efficacy	(70)
FLT3	FLYSYN (mAb)	Phase I/II (NCT02789254)	MRD+ AML (Completed)	FLYSYN	FLYSYN monotherapy is safe and well-tolerated in AML patients with MRD	(71)
FLT3	LY3012218 (IMC-EB10) (mAb)	Phase I (NCT00887926)	R/R AML (Terminated)	IMC-EB10	Terminated due to lack of efficacy	(72)
CD117	Briquilimab (JSP191) (mAb)	Phase I (NCT04429191)	MDS or AML undergoing HSCT (Unknown)	JSP191 + low-dose radiation + fludarabine	Tolerated as non-myeloablative HCT conditioning in adult AML/MDS patients. 93.8% CR (n= 32 AML/MDS), >75% reached MRD negativity	(73)
CD117	MGTA-117 (ADC)	Phase I (NCT05223699)	AML and MDS-EB (Terminated)	MGTA-117	Terminated following sponsor decision	(74)

In a phase-I clinical trial, the safety, tolerability, pharmacokinetics, and preliminary efficacy of a CD3xCD33 BiTE^®^ (bispecific T cell engager developed by Amgen, AMG 330) as monotherapy or in combination with pembrolizumab, a PD-1 inhibitor, in adults with R/R AML were evaluated (NCT02520427, NCT04478695). Treatment with AMG 330, having a molecular weight of approximately 55 kDa and a short terminal half-life of 5.49 ± 1.92 hours, requires extended or continuous administration (for two to four weeks followed by an infusion-free interval between treatment cycles) to improve its therapeutic impact (75). While data on combination therapy with pembrolizumab have not yet been reported, monotherapy results showed that 8 of 60 evaluable patients achieved a complete remission (CR) or CR with incomplete hematologic recovery (CRi), with an overall response rate (ORR) of approximately 13% (CR/CRi). One patient subsequently proceeding to HSCT. Of non-responders, 37% had ≥50% reduction in blasts. Higher disease burden (e.g., higher baseline blast percentage) was associated with increased CRS severity; responses were more frequent in patients with lower-to-moderate burden. Among evaluable patients, the median time to response was 29 days (range: 15–57 days). Treatment-related adverse events (TRAEs) were reported in 94% of patients (72/77). TRAEs of grade 3 and 4 severity were observed in 43 patients (56%) and 23 patients (30%), respectively (75). CRS was reversible and appeared within the first 24 hours of treatment, occurred in 78% of patients (most frequent TRAE); grade ≥3 CRS in ~10–13%. Severity was dose-dependent and correlated with AMG 330 dose level and baseline disease burden. Mitigation included continuous infusion (to allow gradual exposure), dose escalation protocols, and supportive care (e.g., steroids, tocilizumab for severe cases) (75). Although Amgen has not released a detailed public statement on the trial termination, available clinical data suggest that the high incidence of dose-limiting toxicities (DLTs), serious adverse events (SAEs), and insufficient anti-leukemic activity led to the discontinuation of AMG 330 dose-escalation studies.

Next, AMG 673, a CD3xCD33 BiTE^®^ with increased half-life of 21 days, generated by fusing the BiTE^®^ molecule (AMG 330) to the N-terminus of a single-chain IgG Fc region, was tested in a phase-I trial (NCT03224819) (76). A significant reduction of blast counts was found in the BM of 44% of R/R AML patients (12/27 patients). The treatment of the majority of patients in this study was discontinued due to disease progression, with one patient achieving CRi with 85% reduction in blast compared to baseline (76).

AMV564 is a tetravalent bispecific tandem diabody (TandAb) designed to target CD33 and CD3 and has a molecular weight of 106 kDa, resulting in an increased plasma half-life of approximately two to three days (77). Because of the higher avidity, AMV564 was expected to improve T cell-mediated killing efficiency with less toxicity. In a phase-I clinical trial testing of AMV564 for the treatment of R/R AML patients (NCT03144245), no DLTs were reported, a reduction in leukemic blasts was observed in 49% of patients (17/35 patients), and three patients achieved CR, CRi, or partial remission (PR) (77). The most common TRAE was anemia (grade ≥3), generally manageable with appropriate medical intervention.

**Figure 3:**
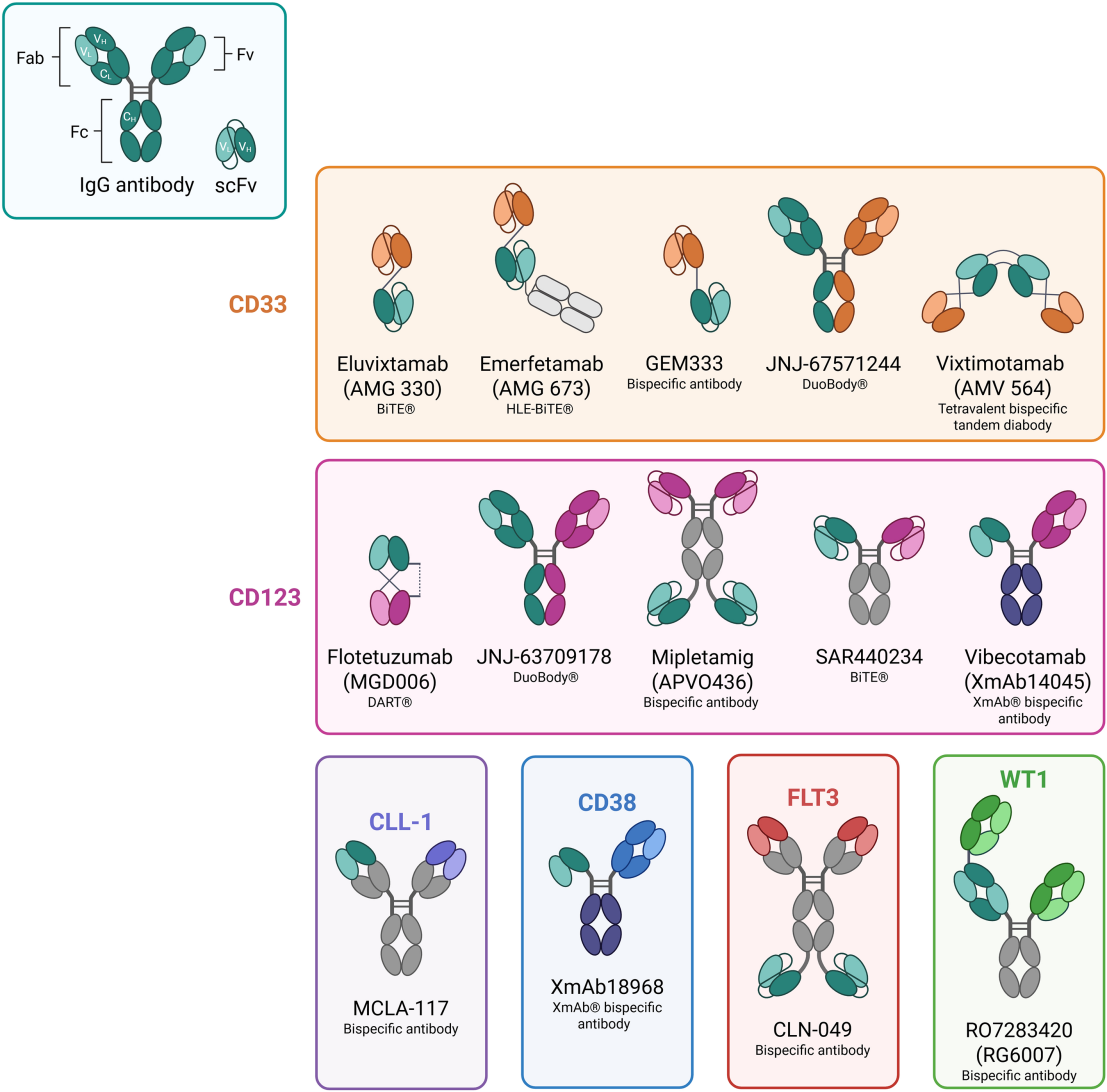
A schematic representation of the structures of bispecific T cell engagers clinically evaluated for AML. Bispecific T cell engager (BiTE); dual-affinity re-targeting antibody (DART); fragment antigen-binding (Fab); fragment crystallizable (Fc); fragment variable (Fv); half-life extended (HLE); immunoglobulin G (IgG); single-chain variable fragment (scFv). Created with BioRender.com

Despite the lower toxicity of AMV564 compared to AMG 673, BTCE-mediated targeted depletion of CD33+ AML cells still needs improvement when used as a single reagent (**Table 2**; **Figure 3**). Janssen Therapeutics has also developed a CD3×CD33 BTCE, JNJ-67571244, which was evaluated in a clinical trial for patients with R/R AML and myelodysplastic syndrome (MDS) (NCT03915379) (78). However, most patients discontinued treatment due to disease progression, and the study was unable to achieve the full treatment dosing. Despite this limitation, the trial provided valuable safety data on JNJ-67571244, which are summarized in **Table 2**.

**Table 2:** Summary of CD3-engaging bispecific antibodies in clinical trials for AML.

Drug name (Manufacturer)	Targets (drug half-life)	Trial phase & status (NCT)	Indication	Therapy	Primary outcome measure	Treatment-related adverse events	Response (CR/CRi/PR)	References
Eluvixtamab (AMG 330) (Amgen)	CD3xCD33 (6 hours)	Phase I – terminated (NCT02520427)	R/R AML, MRD+ AML, or MDS	Monotherapy	DLTs & TEAEs	78% CRS (10% grade ≥3) 30% rash	13% CR/MLFS (8/60) ≥50% BM blast reduction in 37%	(75)
		Phase I – terminated (NCT04478695)	R/R AML	In combination with pembrolizumab	DLTs, TEAEs, &TRAEs	–	–	
Emerfetamab (AMG 673) (Amgen)	HLE-CD3xCD33 (21 days)	Phase I – terminated (NCT03224819)	R/R AML	Monotherapy	DLTs & TEAEs	50% CRS (15/30) 13% CRS grade ≥3	CRi = 1/27 Blast reduction in 44% (12/27)	(76)
GEM333 (AvenCell Europe)	CD3xCD33	Phase I – terminated (NCT03516760)	R/R AML	Monotherapy	MTD & DLTs	No data	No data	–
JNJ-67571244 (Janssen R&D)	CD3xCD33 (63–100 hours)	Phase I – completed (NCT03915379)	R/R AML or MDS	Monotherapy	AEs, DLTs, & ORR	16.2% ≥1 DLT (11/68) 94.1% experienced ≥1 TEAE of grade ≥3 toxicity (64/68)	No activity	(78)
Vixtimotamab (AMV 564) (Amphivena Therapeutics)	CD3xCD33 (2–3 days)	Phase I – completed (NCT03144245)	R/R AML	Monotherapy	AEs, SAEs, & RR	11% anemia grade ≥3 (4/35)	BM blast reduction in 49% (17/35) CR/CRi/PR = 3/35	(77)
Flotetuzumab (MGD006) (MacroGenics)	CD3xCD123	Phase I – completed (NCT05506956)	CD123+ AML post allo-HSCT	Monotherapy	DLTs & MTD	–	–	(79, 80)
		Phase I – active (NCT04681105)	Advanced CD123+ hematological malignancies	Monotherapy	AEs	No data posted yet	No data posted yet	
		Phase II – terminated (NCT04582864)	Relapsed AML and MDS post allo-HSCT	In combination with DLI	CR (MRD), CR, & CRi	–	–	
		Phase II – withdrawn (NCT03739606)	Advanced CD123+ hematological malignancies	Monotherapy	CR, CRi, & CRh	No data	No data	
		Phase II – withdrawn (NCT05063123)	MRD+ AML after 2 cycles of intensive chemotherapy	Monotherapy	CR/CRi without MRD	No data	No data	
		Phase I/II – terminated (NCT02152956)	R/R AML or intermediate-2/HR-MDS	Monotherapy	CR & CRh	81% IRR/CRS (71/88; 8% > grade 3)	ORR; CR, CRh, CRi = 13.6% (12/88)	
JNJ‐63709178 (Janssen R&D)	CD3xCD123 (23–46 hours)	Phase I – completed (NCT02715011)	R/R AML	Monotherapy	DLTs, AEs & SAEs	65-92% TRAEs > grade 3 43.5% any grade CRS	No activity	(81)
Mipletamig (APVO436) (Aptevo Therapeutics)	CD3xCD123 (>7 days in mice ~3 days in monkeys)	Phase I – unknown (NCT03647800)	R/R AML or HR-MDS	Monotherapy	MTD & safety	Grade ≥3 AEs: 25% febrile neutropenia, 18% anemia, 14% hyperglycemia, 11% CRS	Blast reduction (2/28)	(82–84)
		Phase I – withdrawn (NCT04973618)	Elderly or unfit patients with newly diagnosed AML	In combination with ven & aza	Grade 3–4 AEs & SAEs	No data	No data	
		Phase I – recruiting (NCT06634394)	Newly diagnosed AML	In combination with ven & aza	MTD	No data posted yet	No data posted yet	
SAR440234 (Sanofi)	CD3xCD123	Phase I/II – terminated (NCT03594955)	R/R AML, B-ALL, or HR-MDS	Monotherapy	DLTs, OR, DOR, & DFS	–	–	–
Vibecotamab (XmAb14045) (Xencor)	CD3xCD123 (10–25 hours)	Phase I – completed (NCT02730312)	CD123+ hematological malignancies	Monotherapy	TRAEs & MTD/RD	87% TRAEs > grade 3 (105/120) 59.2% CRS any grade 51% hematologic toxicity	9% ORR (10/111)	(85, 86)
		Phase II – active (NCT05285813)	MRD+ AML and MDS after HMA failure	Monotherapy	MRD status & RR	No data posted yet	No data posted yet	
MCLA-117 (Merus NV)	CD3xCLL-1	Phase I – terminated (NCT03038230)	AML	Monotherapy	DLTs	No data	No data	–
XmAb18968 (Xencor)	CD3xCD38	Phase I – terminated (NCT05038644)	R/R CD38+ AML & T-ALL	Monotherapy	DLTs	Grade ≥3: 8% anemia, 15% neutropenia, 23% thrombocytopenia	CR = 2/12	(87)
CLN-049 (Cullinan Oncology)	CD3xFLT3	Phase I – recruiting (NCT05143996)	R/R AML or MDS	Monotherapy	TEAEs & drug PK	50% CRS grade 2 (1/2)	No data posted yet	(88)
RO7283420 (RG6007) (Roche)	CD3xHLA-A2-WT1 (29–84 hours)	Phase I – discontinued (NCT04580121)	R/R AML	Monotherapy	AEs, DLTs, & RP2D	61.3% CRS (9.7% > grade 3) 19.4% TRAEs > grade 5 AEs (1 drug related)	3 out of 42 (7%) achieved CR/CRi	(89)

### 4.2 CD123 – lineage-restricted antigen

CD123, also known as interleukin-3 receptor alpha (IL-3Rα), is essential for the binding of IL-3, a cytokine playing a broad role in hematopoiesis (90). CD123 is found on a wide range of both hematopoietic and non-hematopoietic cells. This includes myeloid precursors, monocytes, basophils, plasmacytoid dendritic cells, as well as epithelial cells in the respiratory and gastrointestinal tracts (91). CD123 is extensively expressed on AML blasts and is notably overexpressed on leukemic stem and progenitor cells in AML, when compared to normal HSPCs (92). Previous studies have shown that the majority of AML patients (50-80% of patients) express high levels of CD123 on at least 80% of their leukemic blasts (93–95). Additionally, it has been shown that elevated CD123 expression correlates with poor prognosis, failure to achieve CR to initial induction chemotherapy, and poor overall survival (96, 97). Taken together CD123 is more specifically overexpressed on AML cells, but its expression on HSPCs and normal tissues has the potential to lead to on-target toxicity. Multiple clinical trials have investigated CD123-targeted therapies in AML. Talacotuzumab, a humanized anti-CD123 mAb, demonstrated preclinical promise and showed biological activity in phase-I. However, subsequent phase-II and phase-II/III trials revealed no significant clinical benefit and notable safety concerns, including infusion-related reactions, cytopenia, and serious infections, ultimately leading to discontinuation of its development in AML and MDS (57, 58). In contrast, pivekimab sunirine, a novel CD123-targeting ADC, remains under active investigation in AML patients (**Table 1**).

Targeted CD123 approaches on AML cells have been evaluated using bispecific T- and natural killer (NK)-cell engagers, dual-affinity retargeting antibody (DART^®^), and trispecific NK cell engagers (58, 79, 82, 85, 98, 99). Several CD3xCD123 BTCEs have entered clinical trials, primarily for AML treatment, such as vibecotamab (XmAb14045; NCT05285813), flotetuzumab (MGD006; NCT04158739, NCT04681105, NCT05506956), JNJ-63709178 (NCT02715011), APVO436 (NCT03647800), and SAR440234 (NCT03594955). While these trials are still ongoing, preliminary data have not yet demonstrated substantial clinical benefits for AML patients (82, 85). For example, in a phase-I study of vibecotamab (NCT02730312) for R/R AML patients seven patients (7/51 patients) achieved CR/CRi or morphologic leukemia-free state (MLFS) (ORR = 14%) treated with higher dose (0.75 µg/kg). Responders had statistically significantly lower absolute blast counts (<25% in blood and bone marrow) compared to non-responders. CRS was the most common TRAE (85% grade 1-2, 15% grade ≥3) but mitigated through a dose-optimized schedule including priming dose (e.g., 1.3 μg/kg) to reduce initial immune activation, followed by step-up and target doses (e.g., 2.3 μg/kg or higher). This approach reduced CRS severity while enabling responses. DLTs (including grade 5 CRS in one case at high target doses) led to holds and refinements (85, 100). In a phase-II study of vibecotamab in measurable residual disease (MRD)-positive MDS/chronic myelomonocytic leukemia (CMML)/AML patients (NCT05285813), 25% of AML patients (3/12) responded to the treatment and achieved MRD negativity after one cycle of treatment. These results suggest potential activity of vibecotamab in the low-blast-burden setting, although the small sample size precludes firm conclusions regarding efficacy at this stage. The responders had prior venetoclax treatment and two of them received HSCT (86). Similarly, monotherapy with APVO436, although showing less potential for TRAEs ≥ grade 3, in a phase-IB trial (NCT03647800) in heavily pre-treated AML/MDS patients (22 R/R AML, 6 R/R MDS) demonstrated blast reduction in two patients (82). Of note, in this study most patients discontinued treatment due to progressive disease.

Flotetuzumab is a DART^®^ with higher affinity for CD123 than CD3, promoting preferential binding of the reagent to leukemic cells. In a clinical study evaluating flotetuzumab in R/R, primary induction failure, and early relapsed AML patients, 13.6% of patients (12/88) achieved CR/CRi/CRh, with a median overall survival (OS) of 10.2 months. A range of TRAEs were observed in this study. Non–infusion related reactions/CRS (non-IRR/CRS) of grade ≥3 included cytopenias, such as anemia (28.4%), thrombocytopenia (20.5%), and reductions in leukocyte and lymphocyte counts (18.2%). IRR/CRS TRAEs were reported in 81% of patients, typically presenting with mild to moderate symptoms; however, 8% experienced grade >3 events. To manage CRS, strategies such as temporary dose reductions or treatment interruptions were employed (80). A phase-I trial of flotetuzumab in pediatric and young patients with R/R AML (ages 3–19 years; NCT04158739) is being conducted to determine the recommended phase-II dose. CRS and capillary leak syndrome were common, with two patients experiencing DLT: grade >3 CRS (1/15) and creatinine elevation (1/15). Overall, CRS occurred in 63% of patients (grades 1–2) and 9% (grade >3). One patient achieved CR but later developed severe CRS, followed by a grade 5 cardiorespiratory event, with autopsy showing CD3+ lymphocyte infiltration and cardiac injury attributed to flotetuzumab. Despite these toxicities, clinical responses were observed in three patients, including two cases of CR/CRi (101). Flotetuzumab is currently being tested in different clinical trials including studies in patients with MRD+ *NPM1*-mutated AML (NCT05063123) and relapsed AML/MDS following allo-HSCT (NCT04582864, NCT05506956). Results from these trials have not yet been reported.

In the subsequent dose-expansion phase, preliminary data from cohorts evaluating APVO436 (at the recommended phase-II dose) in 30 patients with R/R AML (NCT03647800) demonstrated more encouraging activity when used in combination with standard-of-care agents. Composite response rates (CR, CRi, or MLFS) ranged from 20% to 40% across the different combination cohorts. TRAEs >grade 3 were reported in 53% of patients, the most common being anemia (20%), neutropenia (17%), thrombocytopenia (17%), and sepsis (17%). IRR/CRS has been reported for 7% and 23% of patients, respectively, with only one CRS at >grade 3 (83).

Notably, a trispecific NK cell engager targeting CD123, NKp46 and CD16 (SAR443579) is currently under clinical evaluation (NCT05086315) in R/R AML, B-ALL, and high-risk MDS to assess safety and pharmacokinetics (102). JNJ-63709178, a CD3×CD123 BTCE, was evaluated in a phase-I clinical trial (NCT02715011) involving patients with R/R AML. However, treatment with this antibody was associated with an unfavorable safety profile and limited clinical activity, preventing the identification of a recommended phase-II dose (81) (**Table 2**; **Figure 3**).

While CD123 remains an actively explored target through various T cell or NK cell engager platforms, the current CR rates across studies, ranging from approximately 10–25% in R/R AML patients, together with the associated toxicities, suggest that these approaches are still at an early stage of development. Further refinement and optimization will be necessary to improve clinical outcomes in R/R AML. Several potential strategies for advancing T cell engager design and application are outlined in the “Challenges and opportunities” section of this review.

### 4.3 CD47 – leukemia-associated antigen

CD47 is a cell surface glycoprotein, also known as integrin-associated protein, that functions as a ‘don’t eat me’ signal to the innate immune system by binding to signal regulatory protein alpha (SIRPA), an inhibitory receptor on macrophages that blocks phagocytosis (103). CD47 is ubiquitously expressed on normal cells to protect them from phagocytosis. However, its overexpression in various hematological malignancies serves as a key mechanism of immune evasion (104). In AML, CD47 is upregulated on LICs to evade phagocytosis, and this increased expression is often associated with poorer overall survival (105, 106). By inhibiting CD47 expressed on LICs, macrophage-mediated phagocytosis is enhanced, facilitating the clearance and elimination of leukemic cells. Several antibody-based therapies targeting CD47 have been developed and evaluated in clinical trials for AML and/or MDS (59, 64).

Magrolimab, a humanized mAb targeting CD47, has been the focus of extensive clinical investigation. However, most trials were discontinued due to a low probability of meeting the primary endpoint or demonstrating meaningful clinical benefit (60–63, 107). Among the pivotal studies, the ENHANCE-2 and ENHANCE-3 trials are particularly critical, as they represent the comprehensive Phase-III evaluation of magrolimab in AML. The Phase-III ENHANCE-2 trial assessed magrolimab combined with azacitidine in TP53-mutant AML patients, comparing it to azacitidine-venetoclax combination in patients ineligible for intensive treatment or to the intensive standard 7 + 3 chemotherapy in fit patients. Despite promising early-phase data, interim analysis indicated futility, and the final results were negative for both comparisons (61). Similarly, the Phase-III ENHANCE-3 trial tested magrolimab with azacitidine-venetoclax combination in older and unfit AML patients. Although response rates were similar in both arms, fatal adverse events were more common in the magrolimab arm (108). Together, these trials highlight the ongoing challenges in improving outcomes for TP53-mutant AML and patients ineligible for intensive chemotherapy, and raise concerns about the potential limitations of targeting CD47 in these patient populations (**Table 1**).

### 4.4 CD70 – leukemia-associated antigen

CD70 is a membrane-bound ligand that binds to the CD27 receptor, a member of the tumor necrosis factor (TNF)-receptor superfamily (109). Under physiological conditions, CD70 is transiently expressed on antigen-activated B cells, T cells, NK cells, and mature dendritic cells (DC) (110). CD70 and CD27 are co-expressed on AML blasts and HSPCs, but absent on healthy HSCs. This co-expression is associated with activation of HSC gene programs, and elevated soluble CD27 in AML patient sera is linked to poor overall survival (111). On the basis of these observations, CD70 has been evaluated as a therapeutic target in AML, with several CD70-directed agents currently under investigation. Several studies have evaluated CD70-directed therapies in this context. Cusatuzumab (ARGX-110), an anti-CD70 mAb that blocks CD70/CD27 signaling, has demonstrated encouraging clinical activity in AML patients, particularly when combined with azacitidine, and is being further studied in combination regimens (65, 66). In addition, SEA-CD70, a humanized non-fucosylated mAb targeting CD70, has shown strong preclinical efficacy against AML, supporting its potential as a promising CD70-targeted therapy (112) (**Table 1**).

CD70-directed BTCE have been explored using half-life extended BTCE (CD3xCD70) to evaluate its toxicity in cynomolgus monkeys (113). An innovative dual-targeting strategy has also been proposed, where CD70-specific CAR-T cells are engineered to secrete CD3xCD33 BTCEs (114). Despite the innovative approach of targeting CD70, no CD70-targeting BTCEs are currently in clinical trials.

### 4.5 CLL-1 – lineage-restricted antigen

Human C-type lectin-like molecule-1 (CLL-1), also known as CD371, is a type II transmembrane glycoprotein, which is involved in immune regulation as an inhibitory receptor (115). CLL-1 expression is confined to cells of the myeloid lineage and is found on most AML blasts. Additionally, CLL-1 is selectively present on LICs in AML but absent on normal HSCs (116). In addition to its therapeutic potential, CLL-1 functions as a diagnostic marker and has also been recognized for its prognostic value in leukemia. CLL-1 expression levels are associated with disease progression, response to therapy, and overall survival of patients with myeloid malignancies (117). To date, DCLL9718S is the only antibody-based therapeutic, an ADC, targeting CLL-1 that has been tested in clinical trial for AML. However, due to limited tolerability and anti-tumor activity observed in the trial, the program was discontinued (67) (**Table 1**).

### 4.6 TIM-3 – leukemia-associated antigen

T cell immunoglobulin mucin domain-containing protein 3 (TIM-3), also known as CD366, suppresses both innate and adaptive immune responses (118). TIM-3 has been detected on the surface of LICs across multiple AML subtypes, whereas its expression is negligible or absent on healthy HSCs (119, 120). TIM-3 expression on LICs functions as an oncogenic driver by sustaining their proliferative capacity (121, 122). In particular, studies have demonstrated that TIM-3 and its ligand, galectin-9 (Gal-9), form an autocrine signaling loop essential for leukemic stem cell (LSC) self-renewal and the progression of human AML. Consistent with this, it has been shown that serum Gal-9 levels were significantly elevated in AML patients and in mice xenografted with primary human AML cells. Mechanistically, Gal-9 activation of TIM-3 leads to the concurrent activation of NF-κB and β-catenin signaling pathways, both of which are known to support LSC self-renewal (120, 121, 123). Furthermore, elevated TIM-3 expression, at both diagnosis and relapse, has been associated with increased disease aggressiveness and unfavorable clinical outcomes in AML (91, 117, 124, 125). Collectively, its elevated expression and functional role in maintaining LSC survival, highlights TIM-3 as a potential target for AML-directed monotherapy (91). KK2845 (ADC) and Sabatolimab (mAb) are used in TIM-3-targeting therapies for AML and other malignancies. KK2845 is currently in an early phase-I trial, while Sabatolimab has faced challenges with previous trial terminations and withdrawal, but is now recruiting participants in a phase-I/II trial (68) (**Table 1**).

### 4.7 CD38 – leukemia-associated antigen

CD38 is a transmembrane glycoprotein found on lymphoid and myeloid cells, with expression levels that vary according to the cells’ differentiation and activation state (126). Notably, the highest CD38 expression is found on plasma cells, making CD38 a key target for immunotherapy in multiple myeloma (MM) (127). Nevertheless, CD38 is also frequently expressed on AML blasts. Multiple studies have shown that a significant proportion of AML cases express CD38, with positivity rates ranging from 58.2% to 91.7%. However, CD38 expression levels on leukemic blasts vary greatly between patients (128–131). While CD38 is an attractive target in other hematological malignancies, its effectiveness in AML may be more variable, and further studies are needed to fully assess its potential in this context. Despite this uncertainty, CD38 targeting in AML remains an area of interest, with one EMA-approved monoclonal antibody, Darzalex^®^, originally developed for multiple myeloma, is currently being investigated for use in AML (69) (**Table 1**).

Targeting CD38 with BTCEs has been mainly pursued in MM, with XmAb18968, SAR442257, and Bi38-3. XmAb18968, a CD3xCD38 BsAb with reduced FcγR binding to limit off-target activation, is also being evaluated in R/R AML and T-ALL. In a phase-I trial (NCT05038644) enrolling 12 R/R AML patients (77% of whom had adverse-risk, most previously treated with venetoclax-based regimens, and 31% with prior allo-HSCT), treatment with XmAb18968 was associated with grade ≥3 TRAEs, including anemia (8%), neutropenia (15%), and thrombocytopenia (23%). CRS was limited to grade <3 in 62% of patients. Importantly, two patients achieved MRD-negative CR and proceeded to HSCT (87) (**Table 2**; **Figure 3**).

### 4.8 FLT3 – leukemia-associated antigen

FMS-like tyrosine kinase 3 (FLT3), also known as CD135, is a surface expressed member of the class III receptor tyrosine kinase family. Under physiological conditions, FLT3 is expressed in CD34+ HSPCs and is crucial for regulating the survival, proliferation, and differentiation of hematopoietic cells (132, 133). While wild-type FLT3 overexpression occurs commonly in AML blast cells (134, 135), FLT3 mutations are detected in approximately 30% of AML cases, representing the most common genetic alteration in this disease (136, 137). FLT3 is an intensively studied therapeutic target, as both its overexpression and/or mutation are associated with poor prognosis in AML (138, 139). Several antibody-based agents targeting FLT3 have been evaluated for the treatment of AML, but none have demonstrated sufficient efficacy to justify further development (70–72) (**Table 1**).

BTCEs bind to FLT3 on AML blasts independent of mutation status. Preclinical studies have demonstrated that FLT3-targeting BTCEs induce potent T cell-dependent cytotoxicity against FLT3 overexpressed AML cells and confer survival benefits in murine AML models. Notably, targeting FLT3 via BTCE exhibited favorable tolerability in cynomolgus monkeys, with a terminal half-life of 33–50 hours, supporting its translational potential (140). In preclinical models, FLT3-targeted BTCEs showed enhanced AML cell killing in the presence of the costimulatory ligand CD86, a benefit which was less pronounced for FLT3-directed-CAR-T cells (141). Addition of PD-1 blockade and the FLT3 inhibitor quizartinib improved AML lysis by both FLT3-directed BTCEs and CAR-T cells. However, in an AML mouse model, CAR-T cells demonstrated superior survival benefit compared to BTCEs (40 vs. 28 days) (141).

A phase-I clinical study (NCT05143996) is evaluating CLN-049, a CD3xFLT3 BTCE, in R/R AML and MDS patients. Notably, FLT3 expression in BM or peripheral blood was not required for patient eligibility in this trial. Two patients were enrolled, with no unmanageable TRAEs observed. In one patient, CLN-049 levels dropped below the detection limit, while IL-6, IL-8, IL-10, and MCP-1 increased within 6–8 hours post-infusion. In the second patient, CLN-049 was undetectable (88). AMG 427, another CD3xFLT3 BTCE with an extended half-life (33 to 47 hours) showed encouraging preclinical activity. However, its clinical development (NCT03541369) was halted due to CRS, with Amgen providing no additional information on the suspension of patient recruitment (**Table 2**; **Figure 3**).

### 4.9 Promising target antigens

#### 4.9.1 CD117 – lineage-restricted antigen

CD117 (c-KIT), a transmembrane tyrosine kinase receptor for stem cell factor (SCF), is expressed on HSPCs and mast cells, while largely absent from most mature myeloid populations (142, 143). It is detected on leukemic blasts in the majority of AML cases, with expression reported in up to ~70% of patients (144, 145). Functionally, CD117 signaling is critical for the maintenance of normal HSPCs and is also thought to support the survival and persistence of malignant blasts (142, 143, 146, 147). Owing to this dual expression pattern, CD117 has emerged as a compelling target for both conditioning strategies prior to allo-HSCT and for direct therapeutic intervention. In particular, its central role in hematopoiesis and leukemogenesis has prompted investigation into CD117-directed BTCEs, especially in the pre-transplant conditioning setting, although clinical data remain limited and largely confined to early-phase studies.

Briquilimab (formerly JSP191), a CD117-targeting mAb developed by Janssen Therapeutics, has been evaluated in a phase-I trial (NCT04429191) in combination with total body irradiation and fludarabine (TBI/Flu) as non-myeloablative conditioning for allo-HSCT in MRD+ MDS/AML older adult patients. Investigators reported no infusion toxicities and no briquilimab-associated SAEs. Among the 32 evaluable participants (n=13 AML in CR, n=3 AML in relapse, n=16 MDS), 30 patients (93.8%) experienced CR following HSCT. More than 75% of patients with AML in remission and those with MDS reached undetectable MRD levels. A dose of 0.6mg/kg was established as the recommended phase-II dose, with briquilimab clearing within 10–14 days, allowing for successful donor HSPC engraftment. with 90-day post-transplant follow-up, the median donor myeloid chimerism was 99%, and the median total chimerism was 95% (148).

Overall, this trial demonstrated that targeting CD117 with briquilimab, combined with TBI/Flu, represents a safe and well-tolerated conditioning regimen that enables robust HSCT and sustained MRD clearance in older adults with MDS or AML (148). In addition, the CD117-targeting ADC MGTA-117 was evaluated in a phase-I trial (NCT05223699) to assess safety, tolerability, pharmacokinetics, pharmacodynamics, and blast depletion in adults with R/R AML or myelodysplasia with excess blasts. The trial was terminated following a sponsor decision, and no further development has been reported (74) (**Table 1**). Taken together, these results suggest that CD117 is a promising target for further clinical investigation in AML.

Currently, two CD117-targeting BTCEs have been evaluated in preclinical models. These constructs utilize CD3 and CD117 binding domains derived from different sources, yet both demonstrated promising activity in mouse studies (149, 150). In recent preclinical studies in humanized mouse models, we show that CD3xCD117 BTCE treatment results in >99% depletion of healthy CD117+ cells (149). Given its short half-life of approximately two hours and its superior efficacy in eliminating CD117+ cells, this BTCE enabled HSCT shortly after treatment. In humanized mouse models, this BTCE effectively depleted CD117-expressing BM cells, and subsequent HSCT yielded strong donor chimerism. Furthermore, it showed strong depleting activity against AML cells, including LICs (149).

Given the widespread expression of CD117 in normal tissues, including mast cells, concerns remain regarding possible off-target toxicities, such as mast cell depletion or even anaphylaxis. However, JSP 191 has been evaluated in several clinical trials (NCT04429191, NCT02963064, NCT05357482, NCT04784052) without reports of uncontrollable toxicity, suggesting a generally favorable safety profile.

Given the high expression of CD117 in AML, its critical role in leukemia progression, and the encouraging preclinical efficacy of CD117-directed BTCEs, these agents hold strong potential for advancement into clinical trials.

#### 4.9.2 WT1 – leukemia-associated antigen

Wilms’ tumor 1 (WT1) is an intracellular transcription factor that regulates genes involved in nephrogenesis and hematopoiesis (151, 152). Previous studies have shown that WT1 is mutated in approximately 6% to 15% of AML cases, where it acts as a tumor suppressor (153, 154). However, WT1 is overexpressed in AML patients, contributing to leukemogenesis and suggesting its role as an oncogene (155). Several studies have shown that elevated WT1 levels in AML are associated with therapy resistance, an increased risk of relapse, and poor overall survival (151, 156, 157). These characteristics have also led to its use as a marker for MRD detection (158). Because WT1 is an intracellular protein, direct surface targeting is not feasible with conventional antibodies. Instead, TCR-mimetic T cell bispecific antibody (TCBs) such as RO7283420, with a terminal half-life of 29 to 84 hours, have been developed to redirect T cells against a specific WT1-derived peptide (RMFPNAPYL; “RMF”) presented by HLA-A*02:01. Despite clear pharmacodynamic evidence of T cell activation and expansion in early-phase testing, clinical activity has remained modest (3/42 efficacy-evaluable patients achieving CR or CRi; overall response rate of 7%), ultimately leading to early discontinuation of dose escalation and termination of development. DLTs were reported in 11 of 62 patients, including several cases of grade ≥3 CRS. Other grade ≥3 adverse events included stomatitis, myositis, and thrombocytopenia. A total of twelve grade 5 adverse events occurred during the trial, one of which was considered treatment-related (89) (**Table 2**; **Figure 3**). This limited efficacy highlights fundamental biological and translational limitations of peptide-HLA-targeted approaches: therapeutic benefit is inherently restricted to patients expressing the appropriate HLA allele. Primarily HLA-A*02:01, which is present in only ~40–50% of individuals of European ancestry and at lower frequencies in other populations. Even among eligible patients, heterogeneous peptide presentation, potential HLA loss or downregulation on leukemia cells, and the immunosuppressive AML microenvironment can further constrain durable responses (159–161).

Nowadays, preclinical innovation in BTCE design for AML is accelerating, driven by efforts to identify antigens that enhance leukemia specificity while minimizing myelosuppression, a trend anticipated to grow in the coming years. Among these emerging strategies are BTCEs targeting csGRP78, ILT3 (LILRB4), IL1RAP, and TCR−mimetic constructs directed against a Cathepsin G leader−sequence peptide (159, 162–164). Additionally, there is growing interest in dual-specificity BTCEs aimed at enhancing AML cell eradication by simultaneously targeting multiple antigens. Promising constructs under preclinical investigation include CD33/CD123 nanobody-based BTCEs and trispecific formats such as CD3xCD123xFLT3 and CD3xCD28xCD38 (165–167).

## 5 Clinical development of BTCEs beyond R/R AML

Current clinical development of BTCEs in AML has predominantly focused on patients with R/R disease. Most phase-I and early phase-II trials, such as those evaluating AMG 330 (CD3xCD33), vibecotamab (CD3xCD123), APVO436 (CD3xCD123), and RO7283420 (WT1 peptide-HLAxCD3), enrolled heavily pretreated adults, with median ages generally in the sixth to seventh decade where reported (e.g., median age 65.5 years, range 35–84, for RO7283420) (75, 89, 100, 168). Data in special populations remain very limited. Although some trials enrolled patients into their eighth decade of life, no dedicated studies have been conducted specifically in elderly individuals (≥70–75 years), frail patients with significant comorbidities, or those with active post-HSCT relapse; vibecotamab represents a partial exception, as its phase 2 cohorts included patients with prior HSCT (18–42% across disease cohorts), though no subgroup outcomes were formally reported (168, 169). Preliminary experience suggests that BTCEs may be tolerable in older adults when step-up dosing and proactive CRS management strategies, such as prophylactic corticosteroids and tocilizumab, are employed; however, efficacy signals in these subgroups remain modest and often transient (75, 100). Safety concerns are particularly relevant in vulnerable patients, as on-target, off-tumor activity against CD33- and CD123-expressing normal hematopoietic progenitors can lead to prolonged cytopenias and increased susceptibility to infections, as illustrated by RO7283420’s reported pneumonia (28%) and febrile neutropenia (26%) rates (89, 170). Larger, prospective studies specifically designed for elderly, comorbid, or post-transplant patients are urgently needed to better define the safety, efficacy, and optimal positioning of BTCEs in these vulnerable populations.

## 6 Challenges and opportunities

In BTCE therapy, several critical challenges remain that must be addressed to optimize both efficacy and safety. These include the limited availability of tumor-specific markers to distinguish malignant from normal cells, the prolonged half-life of BTCEs needed for some treatment protocols, antigen loss, T cell exhaustion, the risk of severe toxicities, such as CRS, and the emergence of resistance mechanisms like checkpoint inhibitor upregulation. Many of these limitations have been reported and studied in the context of BTCEs for ALL or CAR-T cell therapy, and given their shared strategy of redirecting immune cells toward target cells, they are likely relevant to AML therapy as well. Robust clinical studies are therefore essential to better define these limitations and to guide the development of strategies aimed at improving BTCE efficacy in AML (31).

### 6.1 Off-targeting

Although BTCEs have demonstrated significant advantages, such as their ability to redirect immune cells to target malignant cells with high precision, several challenges and obstacles must be carefully considered, particularly in the context of AML. Unlike certain B-cell malignancies that express highly lineage-restricted antigens (e.g., CD19 or CD20), AML lacks universally expressed, truly leukemia-specific surface antigens. Most current targets are also expressed, at varying levels, on normal hematopoietic cells or HSPCs. This overlap complicates the development of BTCEs that can effectively eradicate leukemic blasts and LICs while minimizing on-target/off-tumor toxicity to normal hematopoiesis. For example, commonly targeted antigens like CD33 and CD123 are also expressed on healthy HSPCs, raising the risk of off-target toxicity. Additionally, target antigens of BTCEs may be expressed on other tissues, increasing the risk of toxicity. For example, CD123 is expressed on endothelial cells, and its levels can be further increased during inflammation. CD123-targeted therapies may further elevate its expression on endothelial cells, potentially triggering a cytokine-driven feedback loop, as has been observed with CD123 CAR-T cell therapy (171).

### 6.2 Preclinical model limitations

A major challenge in the preclinical evaluation of BTCEs for AML is the limited predictive value of currently available animal models for assessing on-target/off-tumor toxicity. Many human-specific BTCEs show poor cross-reactivity with murine or even non-human primate orthologs of the target antigen, making it difficult to accurately evaluate potential toxicities against normal tissues. CD117-targeted BTCEs serve as a pertinent example. The full toxicity profile of these agents, particularly their impact on normal HSPCs, remains incompletely characterized. Humanized mouse models are suboptimal for this purpose due to their inherent immunodeficiency and lack of cross-reactivity between the human-specific CD117-binding domain and murine CD117-expressing tissues. While non-human primate models would provide a more clinically relevant system, this approach is not always feasible when the CD117-binding domain of the BTCE does not cross-react with primate CD117, as reported for certain constructs (150). In contrast, other CD117-directed BTCEs have shown cross-reactivity and are currently undergoing toxicity evaluation in primate models (149).

### 6.3 Half-life

As previously discussed, various design strategies can be employed to increase the half-life of BTCEs, beneficial for sustaining antileukemic activity (172). Notably, the half-life of BTCEs may be further extended when these antibodies remain bound to effector cells, such as T cells, which can act as a reservoir for the antibodies (149, 173). However, this prolonged clearance rate poses a potential challenge, as it may interfere with subsequent interventions, such as HSCT. Residual BTCEs in serum and/or bound to T cells could potentially target and deplete donor-derived HSCs, compromising engraftment and immune reconstitution (149). Sequestering of BTCE on effector cells through their effector cell engaging moiety should always be taken into consideration and strategies to mitigate this need to be employed. For example, after successful depletion of host HSC with BTCE, depleting BTCE “armed” T cells enables efficient engraftment of donor HSC (149). Furthermore, the extended presence of BTCEs may increase the risk of toxicity, including CRS or prolonged immunosuppression, particularly in patients with high disease burden (84, 174, 175). These challenges point to the need for careful optimization of BTCEs design, dosing regimens, and patient selection to balance efficacy and safety in AML therapy.

### 6.4 Tumor microenvironment

The AML TME is highly immunosuppressive and constitutes a major barrier to the clinical efficacy of immune-based therapies, including BTCEs, CAR-T cells, and checkpoint inhibitors. Upon BTCE engagement, AML blasts rapidly upregulate PD-L1 in response to T-cell-derived IFN-γ and TNF-α, as demonstrated with the CD33-targeted BiTE^®^ AMG 330; the resulting PD-1/PD-L1 interactions impair immune synapse stability, reduce T-cell-mediated lysis, and limit overall anti-leukemic activity (176, 177). Cytokine release triggered by T cell redirection further exacerbates resistance by elevating levels of IFN-γ, TNF-α, IL-6, IL-8, and IL-10, which promote checkpoint upregulation and induce feedback suppression of effector T cells. Additional mechanisms include hypoxia-driven metabolic stress, downregulation or loss of HLA class I molecules and antigen-processing machinery on leukemic blasts, and physical barriers that restrict effector T cell infiltration and persistence (178, 179). Moreover, direct contact with bone marrow stromal cells provides niche-mediated protection to AML blasts, diminishing T cell effector functions and destabilizing immune synapses formed by CD3 redirection (180, 181). Although these immunosuppressive mechanisms have not been studied extensively in BTCE-treated AML patients, they provide a plausible explanation for the generally modest and non-durable clinical responses observed with current BTCEs, even in the presence of favorable target antigen expression. Collectively, these TME-driven processes highlight the need for rational combinatorial strategies, such as checkpoint blockade, metabolic modulation, or stroma-targeted agents to overcome this hostile niche and improve therapeutic outcomes.

### 6.5 Loss of antigens

Antigen escape is a well-recognized resistance mechanism in CAR-T cell therapy and can occur through downregulation or loss of target antigens, mutations that alter epitope binding, or the clonal expansion of antigen-negative LICs (182). Although not investigated in AML, given the shared antigen-targeting approach with CAR-T cell therapy, these mechanisms might be relevant to BTCEs as well. Likewise, CD19 loss has been observed in patients receiving blinatumomab (183). Loss of CD19 antigen following blinatumomab therapy arises from multiple interconnected molecular mechanisms. At the genetic and post-transcriptional levels, alternative splicing of CD19 mRNA—particularly deletions of exons 2–4—and dysregulation of mRNA processing mediated by NUDT21 result in reduced or absent expression of functional CD19 epitopes (184, 185). Transcriptional regulation also contributes, as reduced IKAROS (IKZF1) expression is associated with aberrant CD19 splicing, diminished surface expression, and CD19-negative relapse (186). In addition to direct modulation of CD19 expression, lineage plasticity constitutes a major immune-evasion strategy, with documented lineage conversion from B-cell precursor ALL to CD19-negative AML, especially in cases harboring KMT2A rearrangements (187). In contrast, antigen loss as a resistance mechanism to BTCE therapy in AML remains poorly characterized, with limited data on its incidence and underlying mechanisms. While it is biologically plausible that similar processes such as reduced surface expression of CD33, CD123, CLL-1, or WT1 peptide-HLA complexes could contribute to treatment failure, direct evidence from BTCE-treated AML patients remains limited. Most current understanding is extrapolated from CAR-T cell experiences or from observations in other hematologic malignancies. Addressing potential antigen escape will likely require integrated approaches combining predictive biomarkers, consolidative therapies, and novel immunotherapeutic strategies that anticipate and circumvent antigen escape. A logical strategy is to simultaneously target multiple tumor-associated antigens. Antibody-based platforms such as trispecific killer engager (TriKEs) or DARTs are being developed to address this issue. Early examples include constructs directed against CD3xCD70xCD123 currently under clinical evaluation (NCT05673057).

### 6.6 T cell exhaustion

Continuous exposure to BTCEs and persistent T cell stimulation impairs the efficacy of BTCE immunotherapy, primarily due to T cell exhaustion (188), a phenomenon that reduces survival and effector function of T cells. Treatment-free intervals (TFIs) achieved by withdrawing AMG 562 (CD3xCD19), have been identified as an effective strategy to prevent T cell exhaustion (188). Preclinical studies have shown that TFIs promote robust functional reinvigoration and transcriptional reprogramming of T cells, leading to their functional and transcriptional rejuvenation (188). This approach holds potential for application in AML, but the inherent variability in T cell functionality in AML patients remains a critical factor requiring further investigation (189, 190).

### 6.7 Cytokine release syndrome

CRS remains a common challenge in BTCE therapy due to robust T cell activation (191). CRS following BTCE therapy exhibits a biphasic inflammatory pattern. An early phase, occurring within 24–48 hours of BTCE exposure, is driven by T-cell–derived pro-inflammatory cytokines (IFN-γ, TNF-α, GM-CSF), which activate myeloid cells including monocytes, macrophages, and DCs, and initiate downstream immune responses. This is followed by a late phase characterized by myeloid-derived cytokines, particularly IL-6, which correlates with CRS severity and underpins IL-6–targeted therapeutic intervention. CRS amplification results from coordinated interactions among T cells, myeloid cells, and endothelial cells, with myeloid cells serving as key cytokine amplifiers and endothelial activation contributing to systemic inflammatory manifestations (75, 192).

At the molecular level, CRS amplification is driven by interconnected intracellular signaling pathways that regulate cytokine feedforward loops. JAK–STAT and PI3K signaling in myeloid and stromal cells are central to IL-6 production downstream of T-cell–derived cytokines such as IFN-γ and TNF-α (193–195). Preclinical studies demonstrate that targeted inhibition of JAK1/2 or PI3Kγ/δ attenuates IL-6–mediated inflammation while preserving T cell cytotoxic function, highlighting these pathways as promising therapeutic targets for modulating CRS (193, 195).

The severity of CRS in patients receiving BTCE therapy is influenced by multiple factors, including the dosing regimen and administration schedule, as well as the pharmacokinetics and biodistribution affected by CD3 affinity of the BTCE.

Tumor burden is also a critical determinant of CRS severity across BTCE platforms. Experience with blinatumomab in ALL has clearly demonstrated that higher pre-treatment disease burden correlates with increased CRS risk, and cytoreductive strategies, including chemotherapy debulking prior to BTCE initiation, effectively mitigate CRS incidence and severity (191). Although direct data in AML are limited, mechanistic considerations suggest that tumor burden is likewise a critical predictor of CRS risk in AML BTCE therapy. Additionally, patient-specific variables such as comorbid conditions, level of target antigen expression, baseline T cell functionality, and overall inflammatory status play a critical role in determining CRS risk (191, 196–199).

Several strategies can help reduce CRS severity. Stepwise dosing and dose optimization, allowing for gradual T cell engagement, limits the initial cytokine surge. Pharmacological approaches, including cytokine inhibitors, kinase inhibitors like dasatinib, mTOR, and JAK inhibitors, corticosteroids, and supportive care, are also viable options (193, 200–202). For example, in blinatumomab dose-finding studies, a dexamethasone prephase (administered over 5 days) combined with a low starting dose, followed by gradual escalation effectively reduced CRS risk in patients with high leukemic burden (203).

Previous work quantifying native TCR engagement with peptide–MHC complexes (TCR–pMHC) demonstrated the existence of two T cell activation thresholds. Engagement of as few as two TCR–pMHC complexes was sufficient to initiate target cell lysis, whereas the formation of ~10 complexes promoted a fully organized immune synapse and subsequent cytokine secretion. The molecular mechanisms underlying this dual threshold remain to be elucidated (204–206). Similarly, Trinklein et al., reported that BTCEs with CD3-binding domains of varying affinities revealed that even low-affinity CD3 binding could drive potent tumor lysis in murine models, while minimizing cytokine release (207). These findings suggest that the threshold for T cell-mediated cytotoxicity is distinct from that required to trigger CRS, and that it may be possible to dissociate CRS from tumor lysis by employing BTCEs with attenuated CD3 binding affinity (205, 207).

Preclinical studies in non-AML models have shown that while high CD3-affinity BTCEs exhibit strong *in vitro* anti-tumor activity, low-affinity variants achieve comparable efficacy in a humanized mouse models NOD.Cg-Prkdc^scid^ Il2rg^tm1Wjl^/SzJ (NSG) and NOD.Cg-Prkdc^scid^ Il2rg^tm1Sug^/JicTac (NOG) mice with better tolerability and a lower risk of inducing CRS (208, 209).

It is hypothesized that BTCEs with high CD3 affinity may preferentially infiltrate T cell-rich lymphoid tissues rather than the primary tumor site (210). However, as most studies on attenuated CD3-affinity BTCEs have focused on CRS in mouse models, further investigation is needed to assess their enhanced potency and therapeutic benefits in AML patients with varying disease burdens.

Studies demonstrate that CD3ϵ-binding antibodies (e.g., OKT3, UCHT1, L2K, SP34) target unique epitopes, influencing TCR signaling, cytotoxicity, and cytokine release. Therefore, binding of BTCEs to different epitopes of CD3e might also affect T cell activation and signaling strength, and therapeutic outcome (211). These differences can be leveraged to optimize BTCEs for improved efficacy and safety profiles in AML immunotherapy. Structural and spatial characteristics of the target antigen, particularly the epitope’s proximity to the target cell membrane and the antigen’s overall size are determining factors for making stable immunological synapse when using low CD3 affinity BTCE (212, 213). Additionally, the target antigen affinity of BTCEs plays a crucial role in determining their therapeutic potency and risk of off-target effects, which can result in adverse side effects and therefore warrant further investigation. Therefore, BTCE affinity to both target antigen and CD3, expression level of target antigen, are important considerations to mitigate severe CRS.

### 6.8 BTCE resistance mechanisms

Considering the complex pathophysiology of AML and the frequent development of resistance mechanisms, combining BTCEs with current standard-of-care treatments or novel therapeutic agents represents a promising approach to improve clinical outcomes. The strategic integration of BTCE antibodies with conventional AML therapies has the potential to produce synergistic antileukemic effects, overcome therapeutic resistance, and allow for dose reductions of individual drugs, thereby minimizing treatment-related toxicities while maximizing efficacy. Preclinical studies have demonstrated that combining hypomethylating agents, such as azacitidine, with venetoclax and BTCEs elicits synergistic effects in AML models, both *in vitro* and *in vivo* (214). Notably, this synergistic activity appears to be independent of the BTCE target antigen, as comparable synergies have been observed with BTCEs directed against CD33 and WT1 in combination with azacytidine/venetoclax (214). The mechanistic basis for this synergy is partly attributed to venetoclax-induced upregulation of reactive oxygen species (ROS) in T cells, which enhances their effector function and cytotoxic capacity (214, 215). Additionally, another study found that venetoclax pre-treatment dose-dependently increases CD123-directed BTCE-mediated cytotoxicity in AML cells (216). Azacitidine also contributes to this synergy by increasing AML cell sensitivity to T cell-mediated killing, reinforcing the therapeutic rationale for this combination (215). Despite these promising findings, determining the optimal dosing schedule in patients remains a significant challenge, as it is essential to balance efficacy with manageable toxicity.

### 6.9 Immune checkpoint

The upregulation of immune checkpoints, such as PD-L1 and CTLA-4, has been implicated as a resistance mechanism to BTCEs, including blinatumomab, AMG 330, and FLT3-BTCE (140, 176, 217, 218). The increased PD-L1 expression observed in resistant AML cells can be attributed to elevated levels of proinflammatory cytokines, such as IFN-γ, produced by activated T cells, which promote PD-L1 upregulation (176, 219, 220). Studies have demonstrated that post-BTCE treatment the expression of PD-1 and PD-L1 is significantly enhanced compared to baseline levels at diagnosis (176, 218). However, it is still unknown whether this upregulation reflects a byproduct of BTCE-induced T cell activation or represents a direct resistance mechanism employed by leukemic cells. Preclinical mouse studies suggest that combining BTCEs with anti-PD-1/PD-L1 therapy yields greater efficacy than either treatment alone, while also reducing the proportion of anergic T cells (140, 176). These findings are consistent with evidence implicating the PD-1/PD-L1 axis in the immune evasion of AML cells (221). However, this combination has so far been explored in only limited clinical settings. Currently, a phase-I clinical trial is evaluating the combination of flotetuzumab, a CD3xCD123 BTCE, with MGA012, an anti-PD-1 inhibitor, in patients with R/R AML (222).

### 6.10 Emerging strategies and future directions

Innate immune activation represents a compelling strategy to overcome the immunosuppressive AML bone marrow niche. Sensors such as the stimulator of interferon genes (STING) pathway or Toll-like receptor (TLR) agonists can reprogram the marrow into a more proinflammatory, type I IFN-rich environment capable of amplifying BTCE activity. Preclinical studies have shown that cGAMP or small-molecule STING agonists markedly enhance the anti-leukemic cytotoxicity of CD33-directed BTCEs via a positive feedback loop involving target-intrinsic STING/IRF3 signaling, increased secretion of IFN-γ and TNF-α, and upregulation of IFN-stimulated genes that potentiate T cell effector function and persistence (223). These innate immune adjuvants could be rationally combined with various BTCE platforms, including those targeting CD123, CLL-1, or WT1, and may also extend to NK-cell engagers. Improving T cell fitness within the hostile AML microenvironment is another critical avenue. Sustained effector function will likely require multimodal approaches that provide co-stimulation, relieve metabolic suppression, and counteract dominant regulatory populations while leveraging BTCE-mediated reactivation of endogenous leukemia-reactive T cells. Preclinical data indicate that targeted 4-1BB co-stimulation preserves T cell proliferation, cytokine secretion, and metabolic fitness during prolonged BTCE exposure (224). Additionally, inhibition of arginase-mediated arginine depletion can restore T cell proliferation and cytotoxicity, highlighting metabolic intervention as a promising adjunct (225). Notably, BTCEs themselves can convert exhausted marrow T cells into functional leukemia-reactive effectors *ex vivo*, supporting the concept of boosting endogenous T cell quality rather than relying exclusively on cell replacement therapies (226). Shifting BTCE application toward the MRD setting, rather than overt R/R disease, may further improve response depth and durability. This approach benefits from a more favorable effector-to-target ratio and better-preserved T cell function (38). Finally, next-generation BTCE formats, including conditionally activated constructs, multivalent designs, and affinity-differentiated molecules hold substantial promise for widening the therapeutic index by restricting activation primarily to the tumor microenvironment and minimizing systemic toxicity (227, 228). Rationally designed clinical trials that combine STING pathway priming with metabolic and co-stimulatory support, followed by conditionally activated BTCEs in the MRD setting, should be prioritized to maximize both safety and the potential for durable remissions in AML.

## 7 Conclusion

Antibody-based strategies have demonstrated biological activity in preclinical models and early clinical studies in AML, but overall clinical efficacy remains limited. Gemtuzumab ozogamicin is the only FDA-approved antibody-based therapy and provides modest clinical benefit, primarily restricted to specific patient subsets such as those with favorable- or intermediate-risk cytogenetics (particularly core-binding factor AML), while showing limited or no benefit in adverse-risk groups or as monotherapy (44, 229). Broader applicability of antibody-based approaches continues to be constrained by modest single-agent activity, on-target/off-tumor toxicities, and challenges in unselected or R/R populations. Novel BTCE formats and other innovative antibody constructs are being developed with the goal of improving tumor selectivity, optimizing immune synapse formation, and reducing toxicity. Several agents are currently in early-phase clinical trials, where they have shown preliminary anti-leukemic activity; however, responses are generally modest, often transient, and frequently accompanied by significant toxicities such as CRS. Given the limited single-agent activity observed to date, combination strategies incorporating hypomethylating agents, venetoclax, or chemotherapy are being investigated in exploratory early-phase studies. These approaches aim to deepen responses and overcome resistance mechanisms, although definitive evidence of improved outcomes is still awaited from larger trials. While antibody-based therapies, particularly BTCEs, hold conceptual promise, the observed clinical activity remains modest overall. Future research should focus on identifying reliable biomarkers for patient selection, optimizing antibody design and multi-specific formats, exploring rational combinations, and addressing barriers such as the immunosuppressive tumor microenvironment and leukemic stem cell persistence. Such efforts will be essential to expand the therapeutic impact of antibody-based therapies in broader AML populations.

## Glossary

ADC: Antibody-drug conjugates
ALL: Acute lymphoblastic leukemia
Allo-HSCT: Allogeneic hematopoietic stem cell transplantation
AML: Acute myeloid leukemia
BiTE®: Bispecific T cell engager
BM: Bone marrow
BsAbs: Bispecific antibodies
BTCEs: Bispecific T cell engagers
CAR: Chimeric antigen receptor
CDC: Complement-dependent cytotoxicity
CLL-1: C-type lectin-like molecule-1
CMML: Chronic myelomonocytic leukemia
CR: Complete remission
CRh: complete remission with partial hematological recovery
CRi: Complete remission with incomplete hematological recovery
CRS: Cytokine release syndrome
DART®: Dual-affinity retargeting antibody
DC: Dendritic cell
DLTs: Dose-limiting toxicities
Fc: Fragment crystallizable
FcRn: Neonatal Fc receptor
FLT3: FMS-like tyrosine kinase 3
GO: Gemtuzumab ozogamicin
HSCs: Hematopoietic stem cells
HSPCs: Hematopoietic stem and progenitor cells
IgG: Immunoglobulin G
IL-3Rα: Interleukin-3 receptor alpha
IRR: infusion-related reaction
LAAs: Leukemia-associated antigens
LICs: Leukemia-initiating cells
LRAs: Lineage-restricted antigens
LSAs: Leukemia-specific antigens
mAb: Monoclonal antibody
MDS: Myelodysplastic syndrome
MLFS: Morphologic leukemia-free state
MM: Multiple myeloma
MRD: Measurable residual disease
NK cell: Natural killer cell
Non-IRR: Non-infusion related reactions
ORR: Overall response rate
OS: Overall survival
PR: Partial remission
R/R: Relapsed/refractory
SAEs: Serious adverse events
SIGLEC-3: Sialic acid-binding immunoglobulin-like lectin 3
SIRPA: Signal regulatory protein alpha
STING: Stimulator of interferon genes
TanAb: Tetravalent bispecific tandem diabody
TBI/Flu: Total body irradiation and fludarabine
TCR: T cell receptor
TCR-pMHC: TCR engagement with peptide-MHC complexes
TFIs: Treatment-free intervals
TIM-3: T cell immunoglobulin mucin domain-containing protein 3
TLR: Toll-like receptor
TME: Tumor microenvironment
TNF: Tumor necrosis factor
TRAEs: Treatment-related adverse events
TriKE: Trispecific killer engager
WT1: Wilms’ tumor 1
